# Structure of Neuromatous Tumours in Stumps

**Published:** 1843-08

**Authors:** 


*Structure of Neuromatous Tumours in stumps.*—Dr. Bennett exhibited to the Anatomical Society of Edinburgh, February 8, 1843, a neuromatous tumour about the size of a hazel nut, which had been removed from the stump of a patient of his by Mr. Spence. It had caused the individual great agony, which was entirely removed by the operation. On making a section through the tumour, it was found to be composed of a dense white substance, of cartilaginous consistence. Examined microscopically it was seen to be made up of numerous bands of fibres, enclosing an amorphous structure. The bands were composed of from twenty to forty filaments crossing and recrossing each other, and terminating in loops. This filamentous structure differed in its arrangement from any other which Dr. Bennett had hitherto examined. About three-eighths of an inch of the nerve, to which the tumour was attached, had been removed with it, and this presented the same structure as that of the tumour. No remains of nervous tubes could anywhere be detected.

Mr. Spence considered that these tumours, on the ends of divided nerves, were not necessarily productive of pain in the stump. They existed on the cut extremities of nerves in every case, and only give rise to pain when exposed to pressure, when partially implicated in a firm cicatrix, or when thinly covered and thus exposed to the effects of atmospheric changes, or other external causes. When thickly covered by the soft parts it was seldom they gave rise to painful symptoms, though of large size. Mr. Spence, in support of these statements, exhibited a preparation of the nerves from a stump of the leg, where, although a neuromatous tumour existed on every nerve, the only one which had ever given rise to pain had been situated between the cicatrix and end of the fibula. He also showed several drawings of stumps which he had dissected, where the neuromata were very large, and yet had occasioned no pain.—*Lond. and Edin. Month. Journ. of Med. Sci.*, April, 1843, *Amer. Journ.*

